# Supplementary material for: In Vivo Quantification of Myocardial Amyloid Deposits in Patients with Suspected Transthyretin-Related Amyloidosis (ATTR)
Source: J Clin Med. 2020 Oct 27;9(11):3446. doi: 10.3390/jcm9113446 (PMC7693120; doi:10.3390/jcm9113446)
Supplement: Supplementary file 1 [file jcm-09-03446-s001.pdf]

In-vivo quantification of myocardial amyloid deposits in patients with suspected Transthyretin-related Amyoidosis

SUPPLEMENTAL

Figure S1: Comparison of SUVpeak values for Perugini grades 0 and 1 vs 2 and 3.

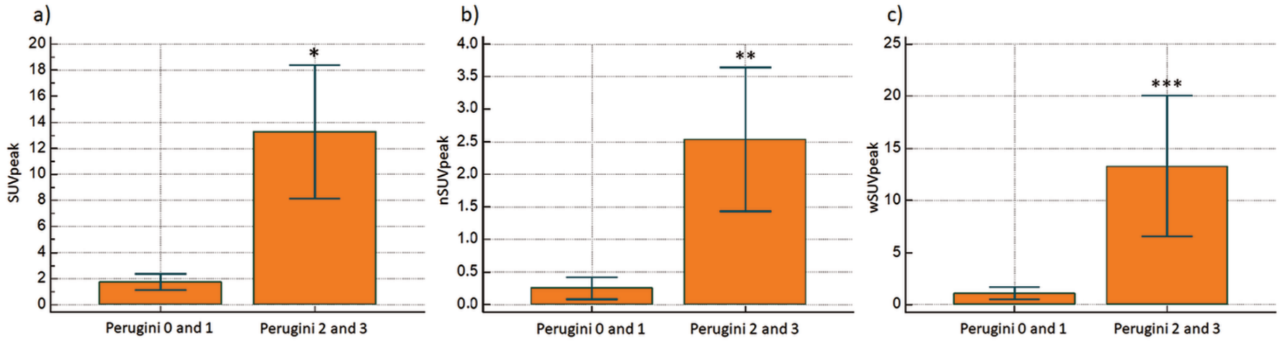

Figure S1: SUVpeak (a), nSUVpeak (b) and wSUVpeak (c) were significantly increased in patients with Perugini grades 2 and 3 compared to patients with Perugini grades 1 and 0 (*p<0.0001 and **p<0.0001, respectively).

Figure S2: Correlation between SUVpeak and nSUVpeak values with Perugini scores.

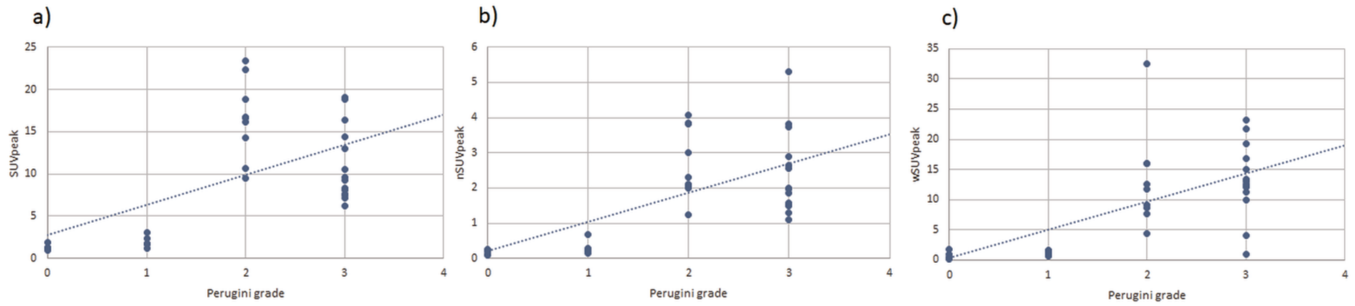

Figure S2: The correlation of SUVpeak, nSUVpeak and wSUVpeak with Perugini grades. All three parametes show a good correlation with Perugini grades (r=0.56; p=0.0009; r=0.62; p=0.0001; r=0.62; p=0.0001; respectively).

Figure S3: Correlation between ECV values and Perugini scores.

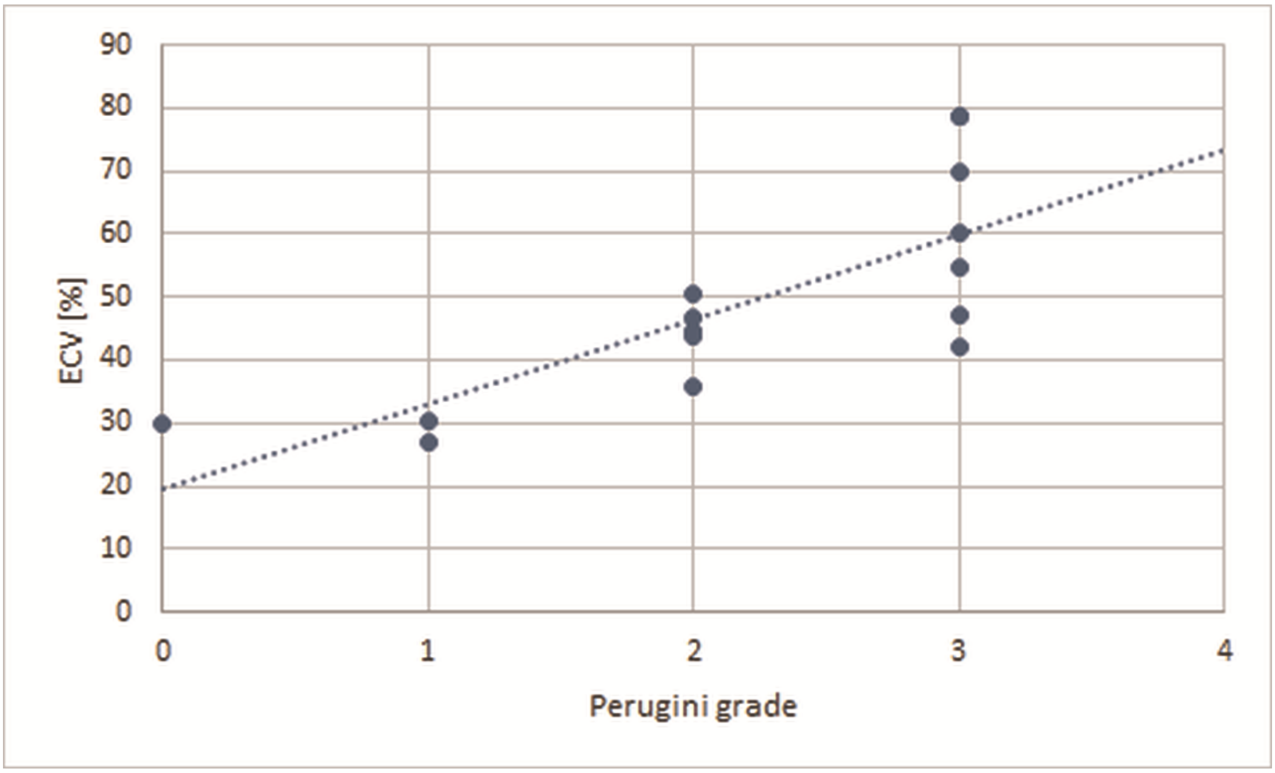

Figure S3: Correlation between ECV values and Perugini scores. ECV shows a good correlation with Perugini scores (r=0.76; p=0.0009).

Figure S4: Correlation of H/CL and H/WB with Perugini grades, SUVpeak, nSUV peak, wSUVpeak and ECV.

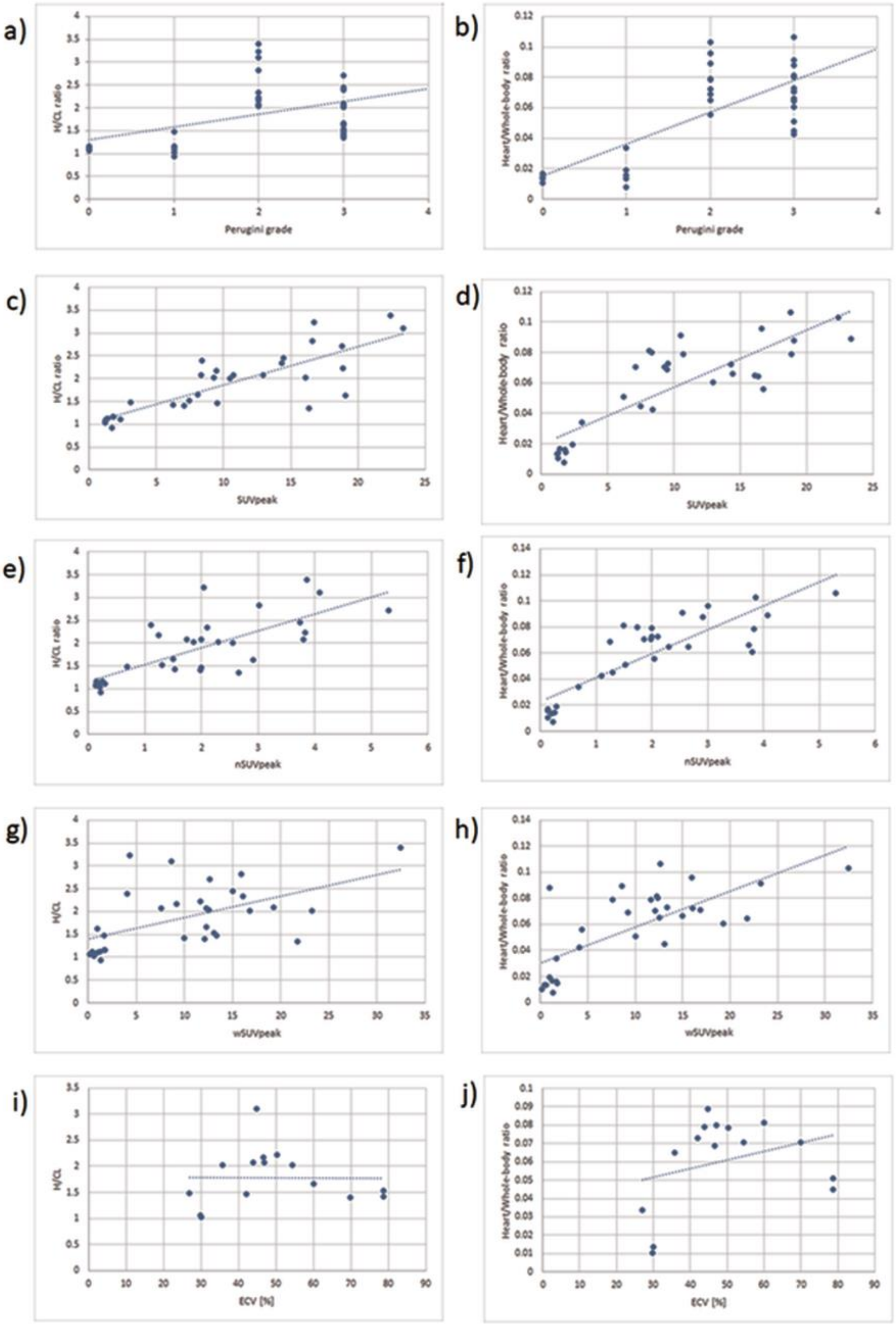

Figure S3: The correlation of semiquantitative H/CL and H/WB with multiple parameters. Both H/CL and H/WB show a good correlation with Perugini grade ($r=0.63$; $p<0.0001$ and $r=0.74$; $p<0.0001$, **a** and **b**), with SUVpeak ($r=0.83$; $p<0.0001$ and $r=0.84$; $p<0.0001$, **c** and **d**), with nSUVpeak ($r=0.75$; $p<0.0001$ and

r=0.85; p<0.0001, **e** and **f**) and with wSUVpeak (r=0.54; p=0.0013 and r=0.72; p<0.0001, **g** and **h**). As shown in **i** and **j** there was no correlation of H/CL and H/WB with ECV ((r=0.005; p=0.99 and r=0.31; p=0.26).
